# Effectiveness of Reverse vs. Traditional Linear Training Periodization in Triathlon

**DOI:** 10.3390/ijerph16152807

**Published:** 2019-08-06

**Authors:** Vicente Javier Clemente-Suárez, Domingo Jesús Ramos-Campo

**Affiliations:** 1 Faculty of Sport Sciences, Universidad Europea de Madrid, 28670 Villaviciosa de Odon, Spain; 2 Grupo de Investigación en Cultura, Educación y Sociedad, Universidad de la Costa, Barranquilla 080002, Colombia; 3 Tritoledo Triathlon Club, 43560 Toledo, Spain; 4 Department of Physical Activity and Sport Science, Sport Science Faculty, Catholic University of Murcia, 30107 Murcia, Spain

**Keywords:** swimming, running, strength, heart rate variability, body composition

## Abstract

The present research aimed to analyze the modification in performance, body composition, and autonomic modulation of reverse and traditional linear training periodization in amateur triathletes. We analyzed running and swimming performance, strength manifestation, body composition, and autonomic modulation before and after a traditional linear training periodization (four weeks of volume-based training plus four weeks of intensity-based training plus two-week tapering), a reverse linear training periodization (four weeks of intensity-based training plus four weeks of volume-based training plus two-week tapering), and a free training control physical active group (10-week free training) in 32 amateur athletes. Independently of the periodization model, the combination of two four-week mesocycles followed by a two-week taper is an efficiency strategy to avoid overreaching, obtaining an increase in parasympathetic modulation. Moreover, both types of training periodization proposed in this study do not modified body composition of amateur triathletes. Also, compared with traditional periodization, reverse periodization efficiently improves horizontal jump performance. Finally, reverse and traditional periodization were an effective strategy to improve running biomechanical, performance, and physiological variables, as well as efficient periodization strategies to improve swimming technical ability, aerobic, and anaerobic swimming performance.

## 1. Introduction

To reach competitive performance, a variety of different training periodization strategies have been applied, varying the distribution of training volumes and intensities during the different training structures of macrocycles, mesocycles, and macrocycles [1,2,3,4,5,6,7,8,9,10,11]. Within these periodization models, the traditional linear periodization based on developing high-volume and low-intensity training during the first periods of the macrocycle, with progressive increases in training intensity and simultaneous decreases in training volumes of the consecutive periods, have been one of the most used [12,13].

Recently, a new periodization model is emerging in opposition to the traditional linear periodization model: The reverse periodization [2,7,14,15,16]. According to the reverse training periodization model, athletes can start their training preparation with high-intensity and low-volume training, while gradually decreasing intensity and increasing volume or, depending on the sport, maintaining intensity and increasing volume during the following training periods [1]. Reverse training periodization has been studied in physical fitness, strength training, swimming, and rowing, obtaining increases in muscular endurance, maximum strength, and endurance performance [9,10,16,17]. The high-intensity interval training, basic for reverse training periodization, showed similar or higher adaptations than high volume of traditional linear training, in muscle buffering capacity and glycogen content, GLUT4, and maximal glucose transport activity in skeletal muscle [18,19,20]. In this line, high intensity interval training (HIIT) produced an increased sympathetic modulation, not negatively effecting cortical arousal and maintaining strength manifestations, but decreasing technical swimming skills if no drills were performed alongside the HIIT training [5,21,22].

A key factor regarding training is efficiency; reverse training periodization has been found to be an effective and time-efficient strategy (since with less training time get the same or larger adaptations) to improve performance mainly for swimming events where the anaerobic threshold is an important performance indicator [15], as well as a model that produces a higher adaptive autonomic response compared with traditional linear periodization [16]. This new model has been studied in different endurance sports such as rowing, running, and swimming [1,2,10,14,23,24,25], but not in triathlon. Based on the literature, we proposed the present research to analyze the modification in running and swimming performance, strength adaptations, body composition, and autonomic modulation of reverse and traditional linear training periodization in triathletes. Changes in running, swimming and horizontal jump performance, autonomic modulation, and body composition were analyzed before and after two 10-week reverse and traditional linear triathlon training programs. We hypothesized that reverse training periodization would achieve higher performance than traditional periodization

## 2. Materials and Methods

### 2.1. Experimental Approach to the Problem

Changes in running, swimming and horizontal jump performance, autonomic modulation, and body composition were analyzed before and after two 10-week reverse and traditional linear triathlon training programs.

### 2.2. Subjects

24 amateur physical active triathletes (11 males: 27.7 ± 5.7 years; 175.2 ± 5.0 cm; 70.6 ± 6.3 kg; and 13 females: 26.8 ± 6.8 years; 164.7 ± 4.6 cm; 58.5 ± 4.1 kg; 5.6 ± 0.4 training sessions/week: 55.2 ± 25.9 min/session; 7.0 ± 1.5 h of training/week; >1 year of experience on triathlon training; competing at national level in sprint and Olympic triathlon distances) participated in the present research. Participants were randomly divided into two different experimental groups:

Reverse periodization (RP) group: They performed 4-week intensity training, 4-week volume training, and 2 weeks of tapering (*n* = 11). Descriptive characteristics of the participants are shown in Table 1.

Traditional periodization (TP) group: They performed 4-week volume training, 4-week intensity training, and 2 weeks of tapering (*n* = 13).

In addition, a physical active control group (CG) was included to control ambient influences and to conduct an experimental design, as previous studies in periodization have used [3]. In this research, they conducted free training without any control by the researchers.

The study design and the procedures employed were in accordance with ethical standards and the Declaration of Helsinki. Each participant was fully informed of the risks associated with the study and they gave a written informed consent before starting the study. If subjects were under 18 years old, written informed consent was obtained from their parents or legal tutor.

### 2.3. Evaluation Test

We evaluated before starting the training programs, after 8 weeks, and after 10 weeks; in the three experimental groups, the following variables we evaluated in this 2-day sequence:

Day 1: Body composition, autonomic modulation by heart rate variability (HRV), and swimming performance;

Day 2: Maximal horizontal jump and running performance.

### 2.4. Body Composition Test

Body composition was assessed with a segmental multifrequency bioimpedance analyzer Tanita BC-600, which uses an eight-point tactile electrode method to take readings from the body. We used the protocol of Clemente-Suarez et al. [26], where participants are informed the day before to come to the test with 1 h of no drink, 2 h of no food intake, with no consumption of drug, medicaments, or caffeine the previous 24 h, and to have urinated and defecated. We conducted the test at the same time, in the same participant order, and in the same place, with a constant temperature and humidity. To carry out the tests, the participants stood upright on foot electrodes on the instrument platform, with legs and thighs apart and arms not touching the torso. They were barefoot and without excess clothing. Four foot electrodes were used, two of which were oval-shaped and two heel-shaped, and prior to testing, both the skin and the electrodes were cleaned and dried, then participants were asked to grip the palm and thumb electrodes (two of each electrode per athlete) according to previous report [27]. Body height was measured using a commercial scale. We analyzed parameters of (I) body mass, (II) body mass index, (III) skeletal muscle mass, (IV) water, and (V) fat percentage.

### 2.5. Heart Rate Variability Test

Before the swimming warm-up, triathletes performed an HRV test using a Polar RS800CX HR monitor (Polar Electro, Kempele, Finland), which lasted for 10 min in a supine, lying in a stretcher in a room with controlled temperature following the procedures of previous research (7). Each participant conducted the HRV test at the same time of the day. The R-R series were analyzed using Kubios HRV software (version 2.0, Biosignal Analysis and Medical Imaging Group, University of Kuopio, Finland). The following HRV variables were assessed: (I) Low-frequency (LF) band/high-frequency (HF) band ratio; (II) percentage of differences between adjacent normal R-R intervals more than 50 ms (PNN50); (III) square root of the mean of the sum of the squared differences between adjacent normal R-R intervals (RMSSD); (IV) mean heart rate; and (V) total power.

### 2.6. Swimming Tests

To analyze swimming performance, we conducted the tests proposed by Gynn [28] to analyze critical speed. Participants performed a 1500 m aerobic swimming standardized warm-up, then a 50 m maximal swimming test, followed by 15 min of rest and a 400 m maximal swimming test. In the 50 m test, we analyzed: (I) Rate of perceived exertion (RPE) with the 6–20 level Borg scale and (II) final heart rate (HR) and (III) speed. In the 400 m test, we analyzed: (IV) Stroke index (V) RPE, (VI) final HR, and (VII) critical speed. Biomechanical parameters were recorded by a slow-motion video camera and analyzed later in a display as previous studies [15].

### 2.7. Maximal Horizontal Jump Test

Subjects performed a standardized warm-up that consisted of 10 min of running (light aerobic). Then, participants performed two maximal horizontal jumps as previously reported [29]. Both jumps were performed with the hands on the waist, to avoid the arm movement inertia, and the best attempt was used for the statistical analysis.

Subjects performed a standardized warm-up that consisted of 10 min of running (light aerobic). Then, participants performed two maximal horizontal jumps as previously reported [29]. Both jumps were performed with the hands on the waist, to avoid the arm movement inertia, and the best attempt was used for the statistical analysis.

### 2.8. Running Test

Running performance was measured by the mean speed of a maximal effort around 2000 m, which is associated with the maximal aerobic speed measured in incremental test conducted in laboratory [30]. After a 10 min aerobic standardized warm up and the maximal horizontal jump test, participants were instructed to run 2000 m at maximal speed in a track surface (temperature 16.1 ± 2.4 °C; 60.2 ± 2.4% humidity). Capillary blood samples (5 μL) for blood lactate concentration ([La-]) analysis were collected from the earlobe immediately after the end of the running test and analyzed using a Lactate Pro analyzer (Lactate Pro, Kyoto, Japan). Variables of (I) stride index (SI = speed x stride length), (II) speed, (III) RPE, (IV) final HR, and (V) [La-] were evaluated. Biomechanical parameters were recorded by a slow-motion video camera and analyzed later in a display as in previous studies [15].

### 2.9. Training Protocol

The randomized design included three different macrocycles, and was conducted after a 4-week training period similar in both training groups:

Reverse training periodization (RP): Composed of a 4-week mesocycle based on high-intensity and low-volume training (Z2 and Z3), 4-week mesocycle based on high-volume and low-intensity training (Z1), and 2-week mesocycle of tapering (combining Z1, Z2, and Z3 with low volume).

Traditional linear training periodization (TP): Composed of a 4-week mesocycle based on high-volume and low-intensity training (Z1), 4-week mesocycle based on high-intensity and low-volume training (Z2 and Z3), and 2-week mesocycle of tapering (combining Z1, Z2, and Z3 with low volume).

Control group training (CG): Free training group during 10-week period (free distribution in Z1, Z2, and Z3); researchers did not any intervene in their training patterns.

**Figure 1:**
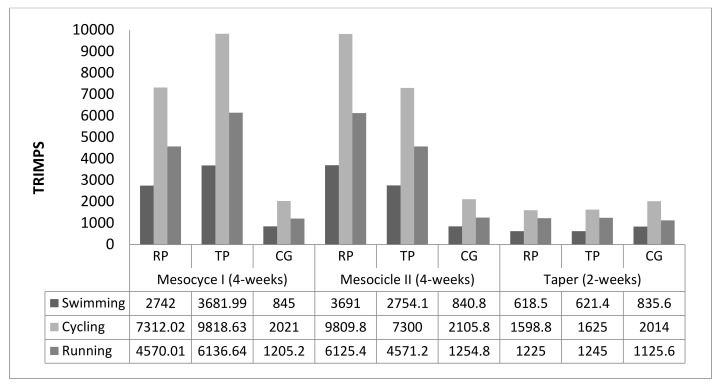
Training impulse (TRIMP) of reverse training periodization group (RP), traditional linear periodization group (TP), and control group (CG) during the three mesocycles of the 10-week macrocycle of training according to the three disciplines.

Training zones were classified according to previous literature in three training zones: Zone 1 (Z1), low-intensity training 65%–80% HRmax; zone 2 (Z2), anaerobic threshold training 80%–95% HRmax; and zone 3 (Z3), high-intensity training >95% HRmax (21). HRmax of participants was obtained in previous training sessions, in order to obtain an individual value of this parameter, not using formulas. Both RP and TP training macrocycles were designed in a low-volume and high-intensity training paradigm as proposed by previous researchers [8,16,31,32,33], just differentiating between them in the distribution of the two first mesocycles. Participants performed a training session of each discipline (swimming, cycling, and running) twice per week. The training load of swimming, cycling, and running sessions conducted by the triathletes was quantified each week using the HR and volume of training to finally assess the training impulse (TRIMP) [34] method (Figure 1).

### 2.10. Statistical Analysis

Data collection, treatment, and analysis were performed using SPSS for Windows statistical package (v.24.0). Descriptive statistics (mean and standard deviation) were calculated. Before using parametric tests, the assumption of normality and homoscedasticity were verified using the Kolmogorov–Smirnov test. A two-way (group × moment) analysis of variance with repeated measures and Bonferroni post hoc was used to investigate differences in variables. The 95% confidence intervals as well as the effect size (ES) are presented in the annexes. ES was tested by Cohen’s D [ES = (Posttest mean—Pretest mean)/Pretest SD]. For all procedures, a level of *p* ≤ 0.05 was selected to indicate statistical significance.

## 3. Results

Table 2 shows the body composition test before and after the 8-week training investigation in the three groups. There were no significant differences in any group between pre- and post-training values. In addition, no significant differences were observed among groups before and after the training program.

The results of the effects on heart rate variability variables (Table 3) before and after the training program showed no significant differences in CG. Also, no significant differences were observed among groups in any moment. However, there were significant differences in RP group in LF/HF, PNN50 between the evaluation performed at 8 weeks and the final evaluation (10 weeks). Moreover, significant differences were found in TP group in total power among the three moments, in LF between basal at 10 weeks and between 8 weeks and 10 weeks’ evaluation and in between 8 weeks and 10 weeks moments. These differences were a main time effect.

Table 4 shows swimming test performance. Both RP and Training groups improved 50 m and 400 m test performance, increasing the stroke index.

In addition, a significant increase in RP from basal values to the end of second mesocycle and the end of taper among groups and in TP at the end of the 8 and 10 weeks in horizontal jump were found (Table 5). Lactate concentration presented an increased value at the end of the 8 weeks of training in RP (Table 5). These differences were a main time effect.

## 4. Discussion

The aim of this research was to analyze the modification in running and swimming performance, strength manifestation, body composition, and autonomic modulation after a reverse and traditional linear training periodization in triathletes. The main findings show that: (i) Both types of training periodization proposed in this study maintain body composition values of amateur triathletes; (ii) both types of training periodization produced a decrease in parasympathetic modulation; (iii) RP and TP are efficient periodization strategies to improve swimming technical ability and swimming performance; (iv) TP negatively affects horizontal jump performance while RP improves it; and (v) biomechanical (length rate, stride rate, and stride index), performance (speed during the second kilometer of the test), and physiological (blood lactate) variables during the 2 km test are specifically improved after both types of periodization (reverse and traditional).

### 4.1. Body Composition

Previous studies showed that training periodization can optimize body composition [14,24]. Body composition has a direct association with physical performance in endurance sports such as triathlon. An excess of fat mass acts as a dead body mass in activities where the body must be repeatedly lifted during running, decreasing performance and increasing energy demands [34]. Also, free-fat mass is an indicator of sports performance [9] because it contributes to the energy production during exercise and provides strength to athletes. However, our results showed no changes in body composition variables after 8 and 10 weeks of training in both types of periodization. This fact contrasted with previous studies showing a decrease of fat mass and an increase in free-fat mass after block periodization [1] or traditional periodization [14,24] in swimmers. In this line, Arroyo-Toledo and de la Rosa [24] confirmed that a traditional periodization training program based on volume is the better option to optimize body composition. However, our data are not in accordance with these findings, but follow the previous results with reverse periodization [14,24] that reported no changes in these variables after training. These discrepancies can be related to the sport modality because in swimming and triathlon, the highest values in fat-free mass may negatively affect buoyance in water and sport economy decreasing performance; also, the baseline values of fat mass and free-fat mass can affect body composition changes [1]. In this way, untrained and recreationally athletes can improve their body composition better than experienced and high-level athletes, whose basal values are much better and more difficult to change [1,26]. Therefore, both types of training periodization proposed in this study maintain body composition values of amateur triathletes.

### 4.2. Heart Rate Variability

Total Power HRV results corroborated the assumption that the increase in autonomic modulation has been associated with increases in athletic performance [35]. This is due to total power significantly increasing performance. Nevertheless, RP did not modify this parameter but increased performance, showing a different autonomic response to training periodization. These apparently conflicting findings might be explained by sport-specific adaptations and the application of the different training protocol [25]. In addition, the decrease in total power achieved by RP was also reported after a marathon and after moderate exercise [36]. The higher parasympathetic autonomic modulation achieved by the TP was consistent with previous research conducted in animal models, where short training sessions were found to be more efficient than high-volume training sessions [37]. This fact was striking considering the same duration of the training sessions of the two experimental groups.

Independently of the periodization model, the accumulation of eight weeks of training produced a decrease in parasympathetic modulation, due to the overreaching status reached by triathletes [30]. It seems that the combination of two four-week mesocycles followed by a two-week taper is an efficient strategy, independently of the training periodization, to avoid overreaching and overtraining, obtaining an increase in parasympathetic modulation (as temporal and frequency HRV domains parameters showed) and sport performance.

### 4.3. Swimming Test

RP significantly increased stroke index after the first eight weeks in the 400 m test. This is a decreased technical efficiency, only observed with the intensity–volume training distribution of RP; nevertheless, after the tapering, the values were not different from baseline. This fact was also evaluated in previous reverse training programs in swimmers and could be attenuated with the implementation of technical swimming drills in the intensity training sessions of the program [15]. The increase in stroke index values in both groups suggests an increase in the mechanical propulsive efficiency of the swimmers, possibly associated with a higher capacity of force production to overcome water resistance [25]. This result showed how RP and TP are efficient periodization strategies to improve swimming technical ability.

Regarding performance in both 50 m and 400 m tests, RP as well as TP increased performance with no significant differences between them. Both groups conducted a training program based on low-volume and high-intensity training as previous authors have shown it to be an efficient tool to improve swimming performance [21,25]. At that point, other authors demonstrated that improvements in swimming performance were not related to training volume but rather to training intensity [38]; that the neuroendocrine and sympathoadrenal responses to exercise were more related to relative training intensity than to absolute intensity [31]; and that high training volumes offers no advantage compared to high-intensity training [33]. At that point, there was no difference between performance before and after the tapering in both groups, which called into question the effectiveness of tapering in these participants. Possibly, in unprofessional triathletes, the simple training drop at the end of each mesocycle could be enough to achieve performance improvements, being able to shorten the macrocycles, increasing the number of main competitions during the season. Notably, previous research [38] found that the triathletes with the worst performance during the baseline can obtain a higher improvement after the training program. Thus, we need to take this fact into consideration because it can justify the lack of differences between group obtained in the present study.

### 4.4. Horizontal Jump Test

Given the well-established interference effects of aerobic endurance exercise on muscle mass and strength and power gains [39], our results suggest that TP negatively affects horizontal jump performance while RP improves it. To the best of our knowledge, no other study has examined the effects of reverse periodization or traditional periodization on jump performance in amateur triathletes. Our results showed that variations in training periodization could interfere in lower body power in both groups. Moreover, reduced jump performance has been reported during periods of heavy intensified training [30] in triathletes and this fact can justify the significant decrease of the jump performance in TP, which performed a higher-intensity load during the last part of the training program. On the other hand, RP group increased the jump performance. This fact is in accordance with Galy et al. [40] which showed an increase in jump performance during the last two weeks of a training program where the load decreased. Therefore, the lower intensity performed by RP at the end of the program can explain the higher values of jump performance. Therefore, RP is an effective periodization model in order to improve horizontal jump height in triathletes. Nevertheless, although previous studies showed how increased intensity periodization produced higher jump performance increases [7], more research is needed to clarify these different results.

### 4.5. Running Test

To our knowledge, this is the first study that has compared a reverse periodization and a traditional periodization in triathletes. A major finding of the present study is that biomechanical, performance, and physiological variables during the 2 km test are specifically improved after both types of periodization. Therefore, according to our results, TP and RP obtained similar results in a 2000 m time trial. This fact is not in accordance with Arroyo et al. [23], who showed that only 10 weeks of reverse periodization increased running performance (400 m and 1000 m) in amateur athletes. Therefore, according to our results, both types of periodization obtained the same results, and this could be due to both groups performing the same training load (TRIMPS) at the end of the training program, as a previous study [39] has shown similar fitness improvements after training with equal volume and load (TRIMPS).

The increased blood lactate after eight weeks of training in both periodization suggests an increased reliance on glycolysis to maintain ATP supply, indicating a greater anaerobic energy release during the test [41]. Therefore, when aerobic metabolism is not capable of meeting ATP demand, the breakdown of phosphocreatine and activation of anaerobic glycolysis can be further elevated to meet the short-term requirements for ATP [42].

Finally, the efficiency of both training periodization conducted in the present study is particularly revealed in a significant increase in stride length and strike index and a decrease in strike rate in both groups. The biomechanical running factors in RP and TP may be associated with possible adaptations linked to an improvement in nervous activation [43]. These adaptations were transferred into running technique due to an improvement in the ground contact times and the stretch–shortening cycle [44] and the increase in stride length as a result of applying more force during foot contact rather than increasing stride frequency [45]. Previous studies observed changes in kinematic running techniques due to fatigue including decreases in stride length [46], however there are no studies that have analyzed the effect of some types of periodization in these parameters. Furthermore, kinematics factors of running technique may be improved by both reverse and traditional periodization.

Compared to previous endurance training research using different types of periodization, this is the first study that compared the effects of RP and TP on running and swimming performance, body composition, horizontal jump height, and autonomic modulation variables in amateur triathletes. We found that both types of training periodization promote similar endurance adaptations in the participants, then both periodization could be and effective training tool for amateur triathletes’ athletes.

We acknowledge some study limitations, specifically related to the lack of cycling performance due to the lack of instrument used to conduct the study. In this line, specific study limitations should be considered for data interpretation, such as that the control group was exposed to lower training load; furthermore, practical recommendations should be restricted to amateur triathletes. Nevertheless, due to the findings in aerobic performance, it can be reasonably suggested from our data that the findings of this study can apply to other athletes such as endurance athletes who may want to optimize their training program. However, it is necessary to conduct more research with endurance athletes to obtain more information about the effectiveness of these types of periodization in people with different fitness levels.

## 5. Conclusions

Reverse and traditional periodization are an effective strategy to improve biomechanical, performance, and physiological variables during a 2 km running test, to improve swimming technical ability, as well as aerobic and anaerobic swimming performance. Independently of the periodization model, the combination of two four-week mesocycles followed by a two-week taper is an efficient strategy to avoid overreaching, obtaining an increase in parasympathetic modulation. Moreover, both types of training periodization proposed in this study maintain body composition values of amateur triathletes. Also, compared with traditional periodization, reverse periodization efficiently improves horizontal jump performance.

## 6. Practical Applications

With the results obtained, triathlon coaches have information to choose two modalities of training periodization; even in the present research, the differences between them were not long, and there is a common factor in them that could help training planification: Both were based on a low-volume and high-intensity paradigm, just modifying the distribution of the loads. This focus on intensity led us to think that this is an important key factor for endurance athletes.

## Figures and Tables

**Table 1:** Descriptive characteristics of the participants at baseline in the three groups, mean ± SD.

Group	n (Male/Female)	Age (years)	Height (cm)	Weight (Kg)	Number of Session/Week	Minutes Per Session	Hours of Training Per Week
RP	11()	25.6 ± 6.8	170.5 ± 6.2	65.4 ± 8.5	5.5 ± 0.2	45.9 ± 24.8	6.9 ± 2.2
TP	13 ()	28.2 ± 9.6	170.5 ± 7.6	66.6 ± 8.7	5.6 ± 0.3	46.3 ± 25.3	7.0 ± 2.1
CG	8 (4/4)	25.9 ± 3.4	166.1 ± 3.9	62.4 ± 5.3	5.8 ± 0.2	48.2 ± 28.2	7.1 ± 2.0

RP: Reverse periodization; TP: Traditional periodization; CG: Control group.

**Table 2:** Data of body composition test before, after 8 weeks of training, and after two weeks of tapering in the three groups, mean ± SD.

	RP (*n* = 11)			TD (*n* = 13)			CG (*n* = 8)			Intergroup Comparations	
	Basal	8 Weeks	10 Weeks	Basal	8 Weeks	10 Weeks	Basal	8 Weeks	10 Weeks	F	P
Weight (Kg)	63.9 ± 7.1	63.8 ± 6.4	63.5 ± 6.5	67.7 ± 9.5	67.8 ± 10.3	67.7 ± 10.0	65.5 ± 8.1	64.6 ± 6.7	64.6 ± 6.7	0.757	0.558
Fat mass (%)	15.8 ± 5.0	15.1 ± 4.8	15.1 ± 5.3	15.6 ± 5.4	15.7 ± 5.5	15.9 ± 5.0	17.0 ± 5.5	16.5 ± 6.8	16.5 ± 6.8	0.289	0.884
Muscle mass (Kg)	51.0 ± 6.3	51.6 ± 6.1	51.4 ± 6.6	54.5 ± 9.6	54.4 ± 9.9	54.2 ± 9.6	50.7 ± 8.0	51.4 ± 8.4	51.4 ± 8.4	0.677	0.611
Bone mass (Kg)	2.7 ± 0.3	2.8 ± 0.3	2.7 ± 0.3	2.9 ± 0.5	2.9 ± 0.5	2.9 ± 0.5	2.7 ± 0.4	2.7 ± 0.4	2.7 ± 0.4	0.573	0.683
Water (%)	61.1 ± 3.2	62.4 ± 3.1	62.4 ± 3.5	61.3 ± 4.0	61.7 ± 3.4	61.5 ± 3.0	60.3 ± 2.7	60.8 ± 3.9	60.8 ± 3.9	0.438	0.781

RP: Reverse periodization; TP: Traditional periodization; CG: Control group.

**Table 3:** Heart rate variability values before, after 8 weeks of training, and after two weeks of tapering in the three groups, mean ± SD.

	RP (*n* = 11)				TP (*n* = 13)				CG (*n* = 8)				Intergroup Comparations	
	Basal	8 Weeks	10 Weeks	Intrag Comp	Basal	8 Weeks	10 Weeks	Intrag Comp	Basal	8 Weeks	10 Weeks	Intrag Comp	F	P
LF (ms^2^)	1294.9 ± 897.2	1837.0 ± 2869.7	2453.3 ± 3128.8		1372.0 ± 1109.5	557.6 ± 293.4	10,696.0 ± 15,625.1	B > 810 > 8	1185.6 ± 1517.8	1865.1 ± 2583.6	1532.1 ± 2665.4		2.740	0.055
HF (ms^2^)	553.7 ± 624.1	284.3 ± 108.2	2725.5 ± 4147.9		427.6 ± 531.4	223.8 ± 149.2	7566.8 ± 13442.1		316.4 ± 242.0	1182.8 ± 2705.5	1211.8 ± 2693.4		1.394	0.271
LF/HF	5.4 ± 6.6	6.1 ± 8.5	1.6 ± 1.5	8 > 10	7.2 ± 10.6	4.0 ± 3.4	2.1 ± 7.7		4.3 ± 4.9	5.0 ± 5.3	2.2 ± 1.3		0.563	0.623
PNN50 %	13.0 ± 15.2	7.7 ± 3.6	23.4 ± 18.9	10 > 8	11.2 ± 14.1	6.3 ± 3.7	18.1 ± 17.9		6.9 ± 3.6	5.8 ± 3.0	7.2 ± 2.3		1.268	0.398
RMSSD (ms)	32.1 ± 16.3	41.0 ± 38.6	72.1 ± 66.3		44.5 ± 42.9	23.7 ± 8.2	110.9 ± 109.3		41.2 ± 42.4	44.8 ± 45.8	42.9 ± 46.1		2.780	0.058
Mean HR (bpm)	69.7 ± 21.8	65.6 ± 9.1	62.3 ± 7.2		69.0 ± 23.2	60.2 ± 7.9	60.2 ± 8.7		65.6 ± 9.1	67.0 ± 7.1	65.5 ± 6.8		1.682	0.1236
TPo (ms^2^)	9935.4 ± 12531.9	21,883.0 ± 23050.9	9024.9 ± 8948.2		4889.6 ± 2889.7	7044.3 ± 9558.1	10,5435.0 ± 14,8253.0	10 > 8 > B	5890.0 ± 5390.9	20,438.6 ± 38,887.5	6234.9 ± 5950.6		1.668	0.237

RP: Reverse periodization; TP: Traditional periodization; CG: Control group; Intrag. Comp.: Intragroup comparation. TPo: Total power; LF: Low-frequency; HF: High-frequency, Norm: Normalized; PNN50: Percentage of differences between adjacent normal R-R intervals more than 50 ms; RMSSD: Square root of the mean of the sum of the squared differences between adjacent normal R-R intervals; HR: Heart rate. B = Basal; 8: Eight-week evaluation; 10: 10 weeks evaluation.

**Table 4:** Swimming performance results before, after 8 weeks of training plus two weeks of tapering in the three groups, mean ± SD.

	RP (*n* = 11)				TP (*n* = 13)				CG (*n* = 8)				Intergroup Comparations	
	Basal	8 Weeks	10 Weeks	Intrag Comp	Basal	8 Weeks	10 Weeks	Intrag Comp	Basal	8 Weeks	10 Weeks	Intrag Comp	F	P
RPE	15.9 ± 2.2	16.6 ± 1.2 ^ǂ^ (0.05)	16.3 ± 1.3 ^ǂ^ (0.05)		16.2 ± 2.1	16.0 ± 2.3 ˠ (0.019)	15.7 ± 1.4 ˠ (0.007)		15.0 ± 2.2	14.3 ± 1.0	14.9 ± 1.0		3.215	0.034
Heart rate (bpm)	177.0 ± 1.4	176.0 ± 1.5	178.0 ± 1.2		176.7 ± 1.3	175.0 ± 1.4	178.0 ± 1.0		173.0 ± 2.5	175.0 ± 1.4	174.0 ± 1.1		1.268	0.298
Speed 50 (m/s)	1.4 ± 0.3	1.4 ± 0.2	1.4 ± 0.3	10 > B	1.3 ± 0.2	1.4 ± 0.2	1.4 ± 0.2	8 > B 10 > B	1.3 ± 0.1	1.3 ± 0.1	1.3 ± 0.1		1.985	0.350
Stroke Index	44.0 ± 11.0	48.9 ± 10.1 ^ǂ^ (0.045)	48.6 ± 10.5 ^ǂ^ (0.035)	8 > B 10 > B	45.9 ± 14.4	49.1 ± 10.6	50.1 ± 11.6	10 > B	37.6 ± 6.3	37.8 ± 6.3	37.7 ± 6.2		3.628	0.041
RPE	17.5 ± 1.3	17.1 ± 1.5	17.5 ± 1.5		16.4 ± 3.0	16.1 ± 2.5	16.8 ± 2.2		14.9 ± 1.9	15.4 ± 1.8	15.1 ± 1.2		1.824	0.214
Critical Speed (m/s)	0.9 ± 0.2	1.0 ± 0.2 ^ǂ^ (0.025)	1.0 ± 0.2 ^ǂ^ (0.032)	8 > B 10 > B	0.9 ± 0.2	1.0 ± 0.2 ˠ (0.039)	1.0 ± 0.2 ˠ (0.039)	8 > B 10 > B	0.8 ± 0.1	0.8 ± 0.1	0.8 ± 0.1		3.864	0.033
Heart rate (bpm)	177.3 ± 1.7 ^ǂ^ (0.01)	175.5 ± 1.3 ^ǂ^ (0.012)	178.2 ± 1.6 ^ǂ^ (0.01)	8 < 10	176.2 ± 3.3 ˠ (0.025)	176.9 ± 2.1 ˠ (0.017)	173.8 ± 1.7 ˠ (0.045)	10 < 8	166.3 ± 9.3	166.3 ± 3.5	166.3 ± 3.5		2.455	0.105

RP: Reverse periodization; TP: Traditional periodization; CG: Control group; Intrag. Comp.: Intragroup comparation; RPE: Rating of perceived exertion. B = Basal; 8: Eight-week evaluation; 10: 10-week evaluation. ^ǂ^ *p* < 0.05 GC vs. RP; ˠ *p* < 0.05 GC vs. TP.

**Table 5:** Data of jump and running performance test before, after 8 weeks of training, and after two weeks of tapering in the three groups, mean ± (SD).

	RP (*n* = 11)				TP (*n* = 13)				CG (*n* = 8)				Intergroup Comparations	
	Basal	8 Weeks	10 Weeks	Intrag Comp	Basal	8 Weeks	10 Weeks	Intrag Comp	Basal	8 Weeks	10 Weeks	Intrag Comp	F	P
Peak Jump length (m)	171.0 ± 20.0	172.4 ± 19.0	172.2 ± 13.6	8 > B 10 > B	170.9 ± 21.5	172.2 ± 25.9	172.1 ± 26.1	8 > B 10 > B	169.5 ± 26.6	169.2 ± 27.3	169.5 ± 25.5		4.317	0.116
Stride Index	3.5 ± 0.3	3.9 ± 0.3	3.7 ± 0.4	8 > B	3.6 ± 0.5	3.8 ± 0.5	3.7 ± 0.6	8 > B	3.5 ± 0.5	3.5 ± 0.5	3.4 ± 0.5		4.258	0.539
Speed (m/s)	4.1 ± 0.5	4.2 ± 0.5	4.2 ± 0.5	8 > B 10 > B	4.2 ± 0.5	4.4 ± 0.5 ˠ (0.001)	4.3 ± 0.5 ˠ (0.003)	8 > B 10 > B	3.7 ± 0.6	3.7 ± 0.4	3.7 ± 0.5		4.279	0.020
RPE	17.7 ± 1.3	17.6 ± 0.8 ^ǂ^ (0.01)	18.6 ± 0.8 ^ǂ^ (0.001)	10 > 8	17.5 ± 1.1	17.6 ± 0.9 ˠ (0.01)	18.1 ± 1.1 ˠ (0.001)		16.6 ± 2.1	15.4 ± 1.6	15.6 ± 1.5	B > 8	1.924	2.149
Final HR (bpm)	192.5 ± 9.4	186.3 ± 11.0	188.6 ± 10.0	B > 8	186.5 ± 5.5	185.3 ± 6.5	186.4 ± 5.9		188.0 ± 20.4	191.4 ± 9.7	191.9 ± 10.5		6.450	0.011
[La-] (mmol/l)	7.4 ± 2.3	10.3 ± 2.0 ^ǂ^ (0.016)	7.0 ± 1.6	10 > B 10 > 8	8.8 ± 2.2	9.5 ± 2.2	7.1 ± 2.4	8 > 10	7.0 ± 1.5	7.4 ± 2.1	7.7 ± 1.6		3.924	0.042

RP: Reverse periodization; TP: Traditional periodization; CG: Control group; HR: Heart rate; Intrag. Comp.: Intragroup comparation; RPE: Rating of perceived exertion. B = Basal; 8: Eight-week evaluation; 10: 10 weeks evaluation. ^ǂ^ *p* < 0.05 GC vs. RP; ˠ *p* < 0.05 GC vs. TP.
